# Editorial: Regulatory Mechanisms of Ca^2+^ Activated Ion Channels and Their Impact on Physiological/Pathophysiological Function 

**DOI:** 10.3389/fphys.2022.876327

**Published:** 2022-04-04

**Authors:** Yoshiaki Suzuki, Susumu Ohya, Wayne R. Giles

**Affiliations:** ^1^ Department of Molecular and Cellular Pharmacology, Graduate School of Pharmaceutical Sciences, Nagoya City University, Nagoya, Japan; ^2^ Department of Pharmacology, Graduate School of Medical Sciences, Nagoya City University, Nagoya, Japan; ^3^ Department of Physiology and Pharmacology, Cumming School of Medicine, University of Calgary, Calgary, AB, Canada

**Keywords:** K_Ca_ channel, BK channel, IK channel, K^+^ current, Ca^2+^ signaling, TMEM16

The main goal of our Research Topic was to identify novel ionic, cellular and tissue level mechanisms that can regulate Ca^2+^-activated K^+^ channel expression and function. An important secondary goal was to summarize current knowledge concerning the consequences of changes in channel activity for initiation and progression of human diseases, and related targets for drug discovery. We also recognized that to achieve these goals it would be necessary to present current knowledge of Ca^2+^-activated ion channel expression and function in intracellular organelles.

We were therefore very pleased with the engagement of the international community in our Research Topic. This resulted in the submission, peer review, and acceptance of 11 very informative reviews or original research articles. The predominant focus that emerged was on the large conductance Ca^2+^-activated K^+^ (BK) channels. Individual contributions focused on the ways in which BK channels form and then function as essential macromolecular signaling complexes, brought about (in part) by their alpha subunits being significant targets for pre- and posttranslational modifications (Sancho and Kyle). This functional theme is taken up and extended in the review by Shah et al., as they provide a succinct summary of what is now known about key aspects of spatially localized or nanodomain signaling complexes that have BK channels as their central element. These complexes can act as an electrophysiological negative feedback regulator for cellular membrane potential and/or agonist-induced changes in intracellular Ca^2+^. An interesting extension of this recent knowledge concerning spatially localized channel expression and related signaling is provided by the clear and concise review by González-Sanabria et al. These authors synthesize and present current knowledge concerning key aspects of the functions of BK channels that are localized to the inner mitochondrial membrane, as well as the outer membrane of the nuclear envelope. When combined, these contributions provide an up-to-date and readable platform of knowledge for beginning to consider the role of BK channels in specific chronic disease pathophysiology; as well as in regulation of cellular homeostasis paradigms (detection and regulation of transient and maintained hypoxia) that need to be recognized and understood as an essential first step for clinical management. For example, the review by Lu and Lee provides a timely summary of the ways in which down regulation of BK channel expression can contribute to vascular dysfunction in the setting of a debilitating chronic disease, diabetes mellitus. The review contributed by Ochoa et al., builds on the fundamental mechanisms through which BK channels can function, both in essential physiological signaling and when dysregulated, to drive progressive pathophysiological sequalae. These authors first focus on hypoxic regulation of BK channels. They summarize basic mechanisms by which BK channels sense cellular oxygen levels, and then undergo hypoxia-induced changes that are specific to either the alpha or one of the beta or gamma subunits of the channel complex. This review concludes with a succinct account of the ways in which the BK channel signaling complex can contribute to the initiation or progression of a number of chronic diseases (including bronchial asthma or obstructive pulmonary disease including sleep apnea). Importantly, the more broadly based review contributed by Jackson reminds the reader of the very broad range of Ca^2+^-activated, or Ca^2+^-dependent, ion channels that are very widely expressed in the mammalian arteriolar vascular system. This review also provides a quite firm reminder that although the most insightful mechanistic studies of ion channel function require a singular focus and a reductionist experimental design; nevertheless, systems level integrative approaches are an absolute requirement for yielding advances in the understanding of essential physiological paradigms, such as the regulation of myogenic tone.

No current summary of Ca^2+^-activated K^+^ channel functionality would be considered to be complete in the absence of presentation of key aspects of what is known about the roles of these channels in neurophysiology. Two articles submitted to this Research Topic provide clear accounts of the roles of Ca^2+^-activated K^+^ channels. Interestingly, both emphasize the role of the integrated functional roles of at least two types of Ca^2+^-activated K^+^ channels in producing CNS phenotypes. McNally et al., provided novel information concerning the ways in which BK channels exhibit significant regulation that is driven by circadian clocks. This important insight is put in context of physiology of the suprachiasmatic nucleus, in part by the authors pointing out that a singular focus on the roles of only BK channels does not suffice. Instead, the contributions of a distinct subset of Ca^2+^-activated K^+^ channels (intermediate conductance) and activation of functionally linked Ca^2+^ channels must be included. For different reasons, and in a separate context, the clear and concise material offered up by Sahu and Turner summarizes the roles of three different Ca^2+^-activated K^+^ channels in hippocampal pyramidal neurons. These authors remind readers of the broadly based importance of being able to fully understand the Ca^2+^-dependent slow afterhyperpolarization phase of the electrophysiological phenotype of CA1 neurons in the hippocampus. Based on their own comprehensive studies Sahu and Turner point out that advances in understanding the function of these neurons requires detailed knowledge of: 1) the profile of changes in intracellular Ca^2+^, 2) spatially localized expression levels of all three subtypes of the Ca^2+^-activated K^+^ channel family, 3) as well as molecular and microanatomical information revealing the ways in which these integral membrane proteins are localized in nanodomains and are linked by molecular chaperones to form signaling complexes. The detailed contribution by Cui returns the emphasis to not only molecular features; but in fact, to atomistic structures. This manuscript illustrates the utility of this approach, when attempting to link some identified BK channel mutations to well recognized neurological disorders.

Our Research Topic was fortunate to receive two papers that provide timely insights into the roles of Ca^2+^-activated Cl^−^ channels. The first (Le et al.) continues our molecular emphasis when presenting known properties of a Ca^2+^-activated Cl^−^ channel denoted TMEM16. Le et al., summarize its major structural features and then focus on its unique gating and regulatory mechanisms. Importantly, this review also establishes that this channel isoform can exhibit specialized enzymatic (flipase) activity. Finally, Wray et al. and her colleagues provide an impactful review that presents some of the major roles of Ca^2+^-activated Cl^−^ channels in key physiological functions of both myometrial and vascular smooth muscles. This Research Topic thus achieves its primary goals while also implicitly making it clear that in this broad, complex and quickly advancing field “more and perhaps the best is yet to come”.

